# Primary central nervous system lymphoma with preceding spontaneous pseudotumoral demyelination in an immunocompetent adult patient: A case report and literature review

**DOI:** 10.3892/ol.2014.2033

**Published:** 2014-04-04

**Authors:** JUNKOH YAMAMOTO, SHOHEI SHIMAJIRI, YOSHITERU NAKANO, SHIGERU NISHIZAWA

**Affiliations:** 1 Department of Neurosurgery, University of Occupational and Environmental Health, Kitakyushu, Fukuoka 807-8555, Japan; 2 Department of Surgical Pathology, University of Occupational and Environmental Health, Kitakyushu, Fukuoka 807-8555, Japan

**Keywords:** multiple sclerosis, steroid, tumefactive, sentinel, biopsy

## Abstract

The rapid disappearance of primary central nervous system lymphomas (PCNSL) following steroid therapy is common; however, the spontaneous regression of PCNSL without any treatment is extremely rare. This study presented a rare case of PCNSL with preceding pseudotumoral demyelination and no previous steroid treatment, and the pitfalls of PCNSL diagnosis were discussed. A 70-year-old healthy male experienced memory and gait disturbances and showed multiple enhanced lesions with perifocal brain edema in the left cerebrum. The patient had no previous symptoms, no chronic lesions and negative oligoclonal immunoglobulin G bands in the cerebrospinal fluid. Histological examination of a brain biopsy specimen revealed predominantly destructive, demyelinating characteristics with infiltration of several T lymphocytes and foamy macrophages resulting in the diagnosis of multiple sclerosis. The patient received steroid therapy and demonstrated gradual improvement, multiple brain lesions had disappeared from the magnetic resonance imaging (MRI)scan two months after the biopsy. However, three months after the biopsy, the condition of the patient deteriorated. MRI indicated a homogeneous enhanced lesion in the right frontal lobe and a second biopsy was performed. Histological examination during the second biopsy revealed a diffuse large B-cell lymphoma. The patient received whole-brain radiation and steroid therapy, however, succumbed eight months following the initial diagnosis. In the current report a comparison between the our case and six previously reported cases is presented.

## Introduction

Primary central nervous system lymphoma (PCNSL) is a rare disease, accounting for 6% of all intracranial tumors and 1–2% of all lymphomas (1,2). Unlike other brain tumors, PCNSL does not warrant radical surgery as the lesions are highly infiltrative (3). PCNSL is a chemosensitive and radiosensitive tumor, thus a stereotaxic biopsy is frequently performed. Early and accurate diagnosis of PCNSL is essential for treatment and prognosis (4).

The temporal disappearance of PCNSL as a result of steroid therapy is well known. Histologically, destructive and demyelinating pseudotumoral characteristics, termed sentinel lesions, are observed following steroid therapy for PCNSL (5). It is difficult to distinguish the sentinel lesions of PCNSL from demyelinating diseases following steroid therapy (5) and few studies have reported the spontaneous radiographic and histological remission of PCNSL (5–8). The current study presents a rare case of PCNSL with preceding pseudotumoral demyelination in an immunocompetent adult that had not received any previous steroid treatment; the pitfalls of PCNSL diagnosis are also discussed. The study was conducted under the auspices of the University of Occupational and Environmental Health Institutional Review Board (IRB; Kitakyushu, Japan) who provided approval for the use of human tissues. The IRB waived the requirement for obtaining informed consent.

## Case report

**Figure 1:**
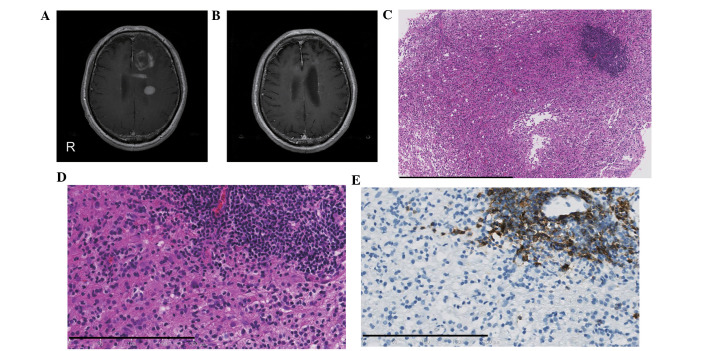
Temporal axial images obtained by brain magnetic resonance imaging and histopathological findings of the tumor specimen obtained during initial surgery. (A) Contrast-enhanced T1-weighted image (CE-T1WI) obtained on admission shows multiple homogeneous and ring-enhanced lesions. (B) CE-T1WI obtained two months after the biopsy shows disappearance of multiple lesions following pulse corticosteroid therapy. (C) Histopathological examination of the biopsy specimen shows demyelinating changes with reactive astrocytes, lymphocytes and foamy macrophages. Focal perivascular aggregation of lymphocytes are demonstrated in the upper right of the image. (D) Magnified view of the boundary area between the perivascular aggregation of lymphocytes and the demyelinated area. (E) Immunohistochemical staining shows that only the lymphocytes with perivascular aggregation were positive for B-cell antibodies (cluster of differentiation 20). (C,D) Hematoxylin and eosin staining. Scale bar: (C) 800 μm and (D and E) 200 μm.

**Figure 2:**
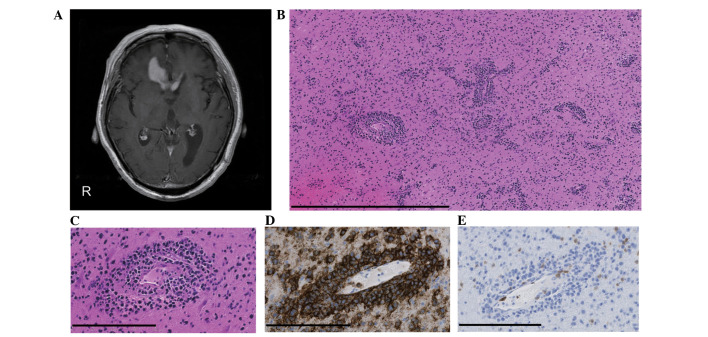
Axial images obtained by brain magnetic resonance imaging and the histopathological findings of the tumor specimen obtained during the second biopsy. (A) Contrast-enhanced T1-weighted image shows new abnormal lesions in the right frontal lobe three months after the initial biopsy. (B,C) Histopathological examination of the specimen shows diffuse infiltrating atypical lymphocytes with perivascular aggregation (hematoxylin and eosin staining). (D) The majority of tumor cells expressed cluster of differentiation (CD) 20. (E) However, the tumor cells were negative for T-cell antibody and CD3. Scale bar: (B) 800 μm and (C–E) 200 μm.

A 70-year-old male with no previous medical concerns experienced memory and gait disturbances and consulted to the University Hospital of Occupational Environmental Health (Kitakyushu, Japan). Neurological examination revealed mild right hemiparesis and dysarthria. The patient’s mini-mental state examination (MMSE) score was 7/30. Magnetic resonance imaging (MRI) showed multiple enhanced lesions with perifocal brain edema in the left cerebral hemisphere (Fig. 1A). Whole-spine MRI, whole-body computed tomography and gascintigraphy showed no abnormalities. In addition, no other abnormal hyperintensity or suspected chronic lesions were detected on MRI. Blood examination, including assessments for determining the levels of soluble interleukin-2 receptor (IL-2R; 388 U/ml) and anti-aquaporin-4, indicated no abnormalities. The serological examination for infectious and collagen diseases was unremarkable. Cerebrospinal fluid (CSF) examination yielded normal results; the CSF showed normal oligoclonal immunoglobulin G bands (OCB) and no myelin basic protein, IL-2R or malignant cells. The patient underwent stereotaxic biopsy after one week. Histological examination of the brain biopsy sample from the left frontal lobe lesion showed myelin destruction with relative sparing of axons, several T lymphocytes and foamy macrophages (Fig. 1C and D). Perivascular aggregation of B-cells was only observed in a small area (Fig. 1E). The patient had no previous symptoms, no chronic lesions on MRI and negative OCB; thus, the condition was diagnosed as multiple sclerosis. The patient gradually improved with pulse corticosteroid therapy. Multiple brain lesions were absent from the MRI scans obtained two months following the biopsy (Fig. 1B) and the MMSE score had increased to 17/30. However, three months after the biopsy, the patient’s condition deteriorated again. MRI revealed a homogeneous enhanced lesion in the right frontal lobe with severe perifocal brain edema (Fig. 2A). Blood examination showed an elevated level of IL-2R (911.0 U/ml). The patient received a brain biopsy with craniotomy. Histological examination of the biopsied lesion revealed diffuse infiltrating B-cells with perivascular aggregation (Fig. 2B–E); therefore the condition was diagnosed as diffuse large B-cell type PCNSL. Following whole brain radiation therapy (45 Gy) and steroid therapy the patient initially showed minimal regression, however, a slow progressive deterioration in neurological and general function subsequently developed. The patient succumbed eight months after the initial diagnosis.

## Discussion

This study presents an unusual case of PCNSL with preceding demyelinating pseudotumoral characteristics in a patient that had not received any previous steroid treatment. Initially, the demyelinating lesions disappeared following steroid therapy and the patient recovered. However, three months after the primary pathological diagnosis, the lesions recurred and were diagnosed as typical PCNSL.

To the best of our knowledge, only six cases of PCNSL with preceding demyelinating pseudotumoral changes and no previous steroid treatment have been published in the literature to date (Table I). The pathogenesis of spontaneous demyelinating changes in PCNSL remains unclear. PCNSL usually develops in the fifth and sixth decades of life, and the male to female ratio for PCNSL is 2:5. A previous study reported that non-Hodgkin’s lymphoma deteriorated during the postpartum period (9). Sudden hormonal changes during pregnancy and menopause may alter the immune system and induce spontaneous regression of PCNSL. Another study reported the case of a male patient with PCNSL who previously had seroconversion of hepatitis B (Tables I and II, case 2); treatment for hepatitis B may have had affected the immunomodulating reaction to PCNSL. However, there was no previous disease in the present case.

Sentinel lesions with PCNSL is a rare occurrence and the incidence rate is currently unknown; however, occurrence may be more frequent than has previously been reported. Distinguishing sentinel lesions with PCNSL from demyelinating diseases is challenging, as demonstrated in the present case where there was no previous medical history of corticosteroid therapy, no typical clinical history (distribution of lesions in space and time) and no other specific laboratory data, including MRI (10). Surgical specimens of lesions obtained from stereotaxic biopsies may be inappropriate for an accurate diagnosis. Furthermore, the biopsy target site affects the pathological diagnosis.

In conclusion, the presence of sentinel lesions indicates remission after corticosteroid treatment and is followed by the diagnosis of typical PCNSL within the next three months to two years. However, PCNSL and demyelinating diseases may respond equally well to steroids (5,7). For a biopsy specimen showing demyelinating lesions, clinicians are required to conduct close clinical and radiological observations to distinguish demyelinating diseases from sentinel lesions due to PCNSL.

## Figures and Tables

**Table I:** Summary of central nervous system lymphoma immunocompetent patients from the literature and the present study without pretreatment of steroids, chemotherapy or radiotherapy.

Case no.	First author (year)	Age (years)/gender	Primary symptoms	Diagnostic modality	Primary site	Primary pathological diagnosis (method)
1	Takekawa H (2008)	68/F	Hypertension	MRI	Bilateral occipital lobe	None (biopsy)
2	Suzuki M (2009)	57/M	General seizure	MRI FDG-PET	Left frontal lobe	Ganglioglioma (biopsy)
3	Alderson L (1996)	49/F	Ataxia, vertigo	CT, MRI	Pons extending to the fourth ventricle	Inflammation (biopsy)
4	Weingarten KL (1983)	69/F	Gait disturbance strange behavior	CT	Bifrontal parasagittal cortical lesions	None
5		69/F	Altered mental status	CT	Right cerebral peduncle extended to caudate nucleus	None
6		60/F	Confusion, obtundation seizure	CT	Left parietal parasagittal cortex	None
7	Present case	70/M	Strange behavior, dementia, right hemiparesis	CT, MRI	Multiple lesions predominantly in the left frontal lobe	Multiple sclerosis (biopsy)

F, female; M, male; MRI, magnetic resonance imaging; FDG-PET, ^18^F-fluoro-deoxyglucose positron emission tomography; CT, computed tomography; DLBCL, diffuse large B-cell lymphoma.

**Table II:** Treatment of central nervous system lymphoma following primary pathological diagnosis.

Case no.	Treatment	Clinical course	Time before progression	Secondary pathological diagnosis (method)	Follow-up prognosis
1	None	Remission of primary lesion, but a later appearance of other multiple lesions	4 months	DLBCL (biopsy)	No information
2	Steroid	Remission, but later appearances of other multiple lesions	24 months	DLBCL (biopsy)	No information
3	Steroid	Remission, but later appearances of right frontal lesion	11 months	DLBCL (biopsy)	No information
4	None	Remission of primary lesions, but later the clinical condition progressively deteriorated	No information	Poorly differentiated malignant lymphoma (autopsy)	Succumbed 4 months after initial presentation
5	None	Remission of primary lesions, then a slow deterioration of the patient’s condition	No information	Malignant undifferentiated lymphoma (autopsy)	Succumbed 3 months after inital presentation
6	None	Remission of primary lesions, patient was treated with steroids and radiotherapy following biopsy	No progression	Malignant lymphoma (autopsy)	Alive 4 years after initial diagnosis
7	Steroid	Remission of primary lesions, but later the clinical condition progressively deteriorated	3 months	DLBCL (biopsy)	Succumbed 8 months after initial diagnosis

DLBCL, diffuse large B-cell lymphoma.
